# Surfaceome Proteomic of Glioblastoma Revealed Potential Targets for Immunotherapy

**DOI:** 10.3389/fimmu.2021.746168

**Published:** 2021-09-27

**Authors:** Mélanie Rose, Tristan Cardon, Soulaimane Aboulouard, Nawale Hajjaji, Firas Kobeissy, Marie Duhamel, Isabelle Fournier, Michel Salzet

**Affiliations:** ^1^ Université Lille, Inserm, CHU Lille, U1192, Laboratoire Protéomique, Réponse Inflammatoire et Spectrométrie de Masse (PRISM), Lille, France; ^2^ Breast Cancer Unit, Oscar Lambret Center, Lille, France; ^3^ Department of Biochemistry and Molecular Genetics, Faculty of Medicine, American University of Beirut, Beirut, Lebanon; ^4^ Institut Universitaire de France, Paris, France

**Keywords:** surfaceome proteomic, glioblastoma, immune therapy, surface proteins, mutated proteins, drugs, clinical trials

## Abstract

Glioblastoma (GBM) is the most common and devastating malignant brain tumor in adults. The mortality rate is very high despite different treatments. New therapeutic targets are therefore highly needed. Cell-surface proteins represent attractive targets due to their accessibility, their involvement in essential signaling pathways, and their dysregulated expression in cancer. Moreover, they are potential targets for CAR-based immunotherapy or mRNA vaccine strategies. In this context, we investigated the GBM-associated surfaceome by comparing it to astrocytes cell line surfaceome to identify new specific targets for GBM. For this purpose, biotinylation of cell surface proteins has been carried out in GBM and astrocytes cell lines. Biotinylated proteins were purified on streptavidin beads and analyzed by shotgun proteomics. Cell surface proteins were identified with Cell Surface Proteins Atlas (CSPA) and Gene Ontology enrichment. Among all the surface proteins identified in the different cell lines we have confirmed the expression of 66 of these in patient’s glioblastoma using spatial proteomic guided by MALDI-mass spectrometry. Moreover, 87 surface proteins overexpressed or exclusive in GBM cell lines have been identified. Among these, we found 11 specific potential targets for GBM including 5 mutated proteins such as RELL1, CYBA, EGFR, and MHC I proteins. Matching with drugs and clinical trials databases revealed that 7 proteins were druggable and under evaluation, 3 proteins have no known drug interaction yet and none of them are the mutated form of the identified proteins. Taken together, we discovered potential targets for immune therapy strategies in GBM.

**Graphical Abstract:**
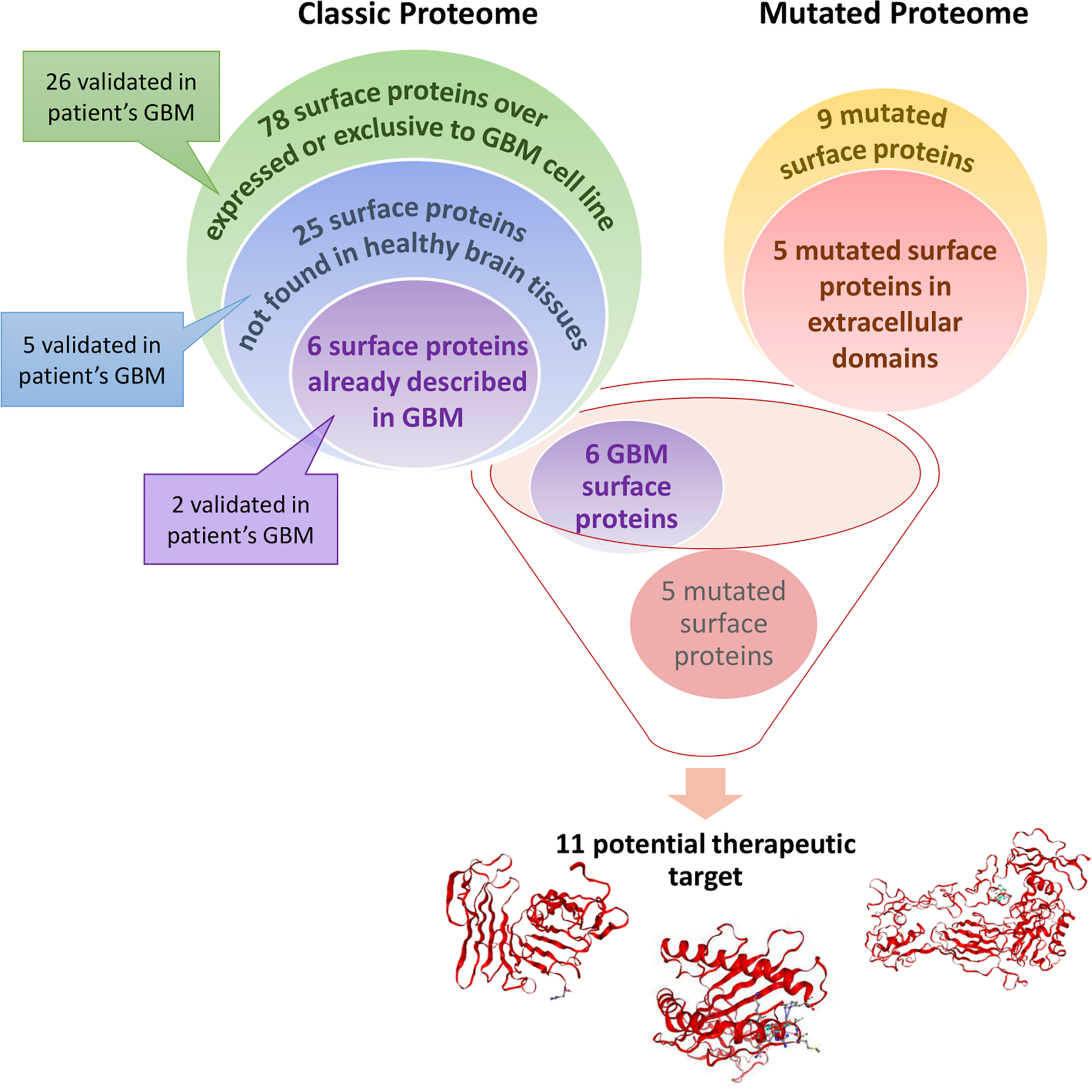
Glioblastoma (GBM) cell line surface proteome was compared to healthy astrocytes cell line surface proteome. A total of 78 surface proteins are identified as over-expressed or exclusive to the GBM cell line. According to Human Protein Atlas, 25 of these surface proteins are not found in healthy brain tissues. Among these 25 surface proteins, 6 are already described in (GBM) and could be potential therapeutic target. Some of the proteins described above have also been found in a cohort of 50 GBM patients. Moreover, we identified 9 mutated proteins only expressed by GBM cell lines in which 5 are mutated in their extracellular domains which is interesting for targeted therapy. Thus, these 11 proteins could be potential therapeutic targets for CAR-based immunotherapy or mRNA vaccine strategies.

## Introduction

Glioblastoma represents the main malignant primary brain tumor with an incidence of 3.22 per 100,000 population (1). The prognosis is poor with median survival estimated at 16 months in clinical studies if treated with at least a near-total resection (QTR) and followed by the Stupp protocol (SP) (2–8) and around 12 months in contemporary population-based studies (9). Approximately 7% of patients survive more than 5 years after diagnosis (1). Favorable therapy-independent prognostic factors include lower age and higher neurological performance status at diagnosis. Furthermore, low postoperative residual tumor volume has been associated with improved outcomes. Moreover, GBM treatment options such as oncogenic signaling pathways including RTK/Ras/PI3K (88%), p53 (87%) and pRB signaling pathways (78%), or VEGF-targeting monoclonal antibody Bevacizumab, DNA alkylating agents such as lomustine and carmustine implants, and the checkpoint blockade inhibitor have been underwhelming in GBM (10, 11). For immunotherapy, resistance is due to strong local immunosuppression and to the difficulty of identifying highly specific tumor antigens. Very few GBM-specific antigens have been identified so far and they show a very limited potential as targets for immunotherapy.

In this context, several strategies tried to identify surface protein targets. In fact, 10 to 20% of all genes in the human genome encode cell surface proteins and due to their subcellular localization, these proteins represent excellent targets for cancer diagnosis and therapeutics. Recent studies have integrated transcriptomic and proteomic data for such a purpose in GBM (12). 395 genes were classified as coding for surface proteins and 6 were identified with high confidence i.e. HLA-DRA, CD44, SLC1A5, EGFR, ITGB2, PTPRJ, which are upregulated in GBM (12). Bausch-Fluck et al. (13) have developed a mass spectrometric-derived cell surface Atlas (CSPA) integrating GBM cell lines (13, 14). Thus, it is now possible to compare new surfaceome proteomic dataset provided experimentally to CSPA database and from mutated peptides from XMan v.2 database (15). In this context, we compared astrocytoma GBM cell lines to normal astrocytes cell line. We could identify 11 specific potential targets for GBM including 5 mutated proteins (PLAUR, ITGB3, and MHC I proteins). The specificity of these proteins to GBM has been validated using the human protein atlas and a cohort of 50 GBM patients.

## Materials and Methods

### Experimental Design and Statistical Rationale

Shotgun proteomics experiments were conducted in biological triplicates (n=3). For the proteomics statistical analysis, extracted proteins presenting as significant by the ANOVA test analysis were used (p-value < 0.05). Normalization was achieved using a Z-score with matrix access by rows.

### Chemicals and Reagents

Water (H^2^O), formic acid (FA), acetonitrile (ACN), and trifluoroacetic acid (TFA) were obtained from Biosolve B. V. (Valkenswaard, Netherlands). DL-dithiothreitol (DTT), Hydrochloric acid (HCl), and Triton X-114 were purchased from Sigma-Aldrich (Saint-Quentin Fallavier, France). Trypsin was purchased from Promega (Charbonnieres, France). Tris was purchased from Interchim (Montluçon, France). EZ-Link Sulfo-NHS-SS-Biotin, Streptavidin UltraLink Resin (Pierce), Sodium chloride (NaCl), Dulbecco’s Modified Eagle’s Medium (DMEM), DMEM high glucose GlutaMAX™ Supplement, heat-inactivated fetal bovine serum (FBS), trypsin, phosphate buffer saline (PBS), penicillin and streptomycin were purchased from Thermo Fisher Scientific (Massachusetts, USA). U-87 MG (ATCC^®^ HTB-14™) cell lines were purchased from ATTC (Manassas, Virginia, USA). Immortalized Human Astrocytes (Ref: P10251-IM) and astrocyte medium were obtained from Innoprot (Derio, Spain). Human NCH82 stage IV glioma cells were obtained from Dr Regnier-Vigouroux.

### Cell Culture

Human glioma cell line NCH82 and U-87 MG were grown in DMEM and DMEM high glucose GlutaMAX™ Supplement respectively supplemented with 10% FBS, 1% L-glutamine (2 mM), and 1% penicillin/streptomycin (100 units per ml). Immortalized human astrocytes were grown in an appropriate medium purchased from Innoprot. All cell lines were cultured at 37°C in a humidified atmosphere (5% CO2).

### Cell Surface Protein Biotinylation and Triton X-114 Phase Partitioning

The same amount of cells (20.10^6^) were plated on sterile 15 cm dish and cultured until they reached ≃ 80% confluence. Cells were washed three times with ice-cold PBS and incubate 30 min at room temperature with 0.25 mg/ml EZ-Link Sulfo-NHS-SS-Biotin with gentle agitation. The reaction was quenched with 50 mM Tris-HCl and cells were washed two times with ice-cold PBS. Cells were then scraped and resuspended in aqueous 2% (w/v) Triton X-114, containing 10 mM Tris–HCl, pH 7.5, and 150 mM NaCl and incubated 30 min at 4°C with frequent vortexing. Cell debris was removed with centrifugation at 10,000 x g at 4°C for 10 min. Supernatants were collected and incubated for 15 min at 37°C to achieve phase partitioning. The suspension was then centrifuged at 5,000 x g at 25°C for 30 min. The upper aqueous phase was discarded, and the lower detergent phase was carefully collected.

### Purification of Biotinylated Proteins

Prior to experiments, the beads were pre-equilibrated with lysis buffer. The lower detergent phase was incubated with 40 μl of a 50% slurry of pre-equilibrated Streptavidin UltraLink Resin (Pierce) for 3 hrs at 4°C on a rotating well. After 6 washes with 50 mM ammonium bicarbonate buffer, biotinylated proteins were eluted with 50mM DTT 30 minutes at 50°C.

### Protein Digestion

The proteins were digested with 1 µg Trypsin (Promega) overnight at 37°C. The digestion was stopped with 0.5% TFA. The samples were desalted using ZipTip C-18 (Millipore) and eluted with a solution of ACN/0.1% TFA (7:3, v/v). The samples were dried with SpeedVac and resuspended in 20 µL of ACN/0.1% formic acid (0.2:9.8, v/v) just before processing using LC-MS/MS. Experiments were done in biological triplicate (n=3).

### LC-MS/MS Analysis

Mass spectrometry proteomics analysis of digested proteins was performed using a nano Acquity UPLC system (Waters) coupled with the Q-Exactive Orbitrap mass spectrometer (Thermo Scientific) *via* a nanoelectrospray source. The samples were separated using online reversed-phase, using a preconcentration column (nanoAcquity Symmetry C18, 5 µm, 180 µm x 20 mm) and an analytical column (nanoAcquity BEH C18, 1.7 µm, 75 µm x 250 mm). The peptides were separated by applying a linear gradient of acetonitrile in 0.1% formic acid (5%-35%) for 2h, at a flow rate of 300 nl/min. The Q- Exactive was operated in data-dependent mode defined to analyze the ten most intense ions of MS analysis (Top 10). The MS analysis was performed with an m/z mass range between 300 to 1 600, resolution of 70,000 FWHM, AGC of 3e6 ions, and maximum injection time of 120ms. The MS/MS analysis was performed with an m/z mass range between 200 to 2,000; AGC of 5e4 ion; maximum injection time of 60 ms and resolution set at 17,500 FWHM.

### Protein ID and Data Analysis

Proteins were identified by comparing all MS/MS data with the proteome database of the complete reviewed proteome of Homo sapiens (Uniprot, release November 2020; 20,370 entries), using the MaxQuant software version 1.6.10.43 (16, 17). Trypsin specificity was used for the digestion mode with two missed cleavages. N-terminal acetylation and methionine oxidation were selected as the variable modifications. For MS spectra, an initial mass tolerance of 6 ppm was selected, and the MS/MS tolerance was set to 20 ppm for HCD data (18). For identification, the FDR at the peptide spectrum matches (PSMs) and protein level was set to 0.01, and a minimum of 2 peptides per protein in which 1 was unique. Relative, label-free quantification of proteins was performed using the MaxLFQ algorithm integrated into MaxQuant with the default parameters. This algorithm performed the normalization of the MS-data in the total peptide ion signals. The normalization factors is calculated between the different cell condition and replicates (19). Analysis of the proteins identified was performed using Perseus software (http://www.perseus-framework.org/) (version 1.6.5.0) (20). The file containing the information from identification was used with hits to the reverse database, and proteins identified with modified peptides and potential contaminants were removed. Then, the LFQ intensity was logarithmized (log2[x]). Categorical annotation of rows was used to define different groups depending on the cell line. Multiple-samples tests were performed using an ANOVA test with a p-value of 0.05 and preserved grouping in randomization. The results were normalized by Z-score and represented as hierarchical clustering. Functional annotation and characterization of identified proteins were obtained using STRING (version 11.0, http://string-db.org). Surface proteins were then identified with the lists of surface proteins provided by the cell surface protein atlas (CSPA) (13) and the list of predicted surfaceome proteins (14). Gene Ontology enrichment allows the identification of some additional surface proteins. To identify surface protein already described in GBM we grouped the surface proteins described in the CSPA of primary brain tumor and GBM cells and in the CSPA of GBM cell lines LN18, LN229, U251-MG, U87-MG, and T98G GBM cell line (13). Venn diagram analysis was performed using “the InteractiVenn” (21).

### Sub-Network Enrichment Pathway Analysis

Using Elsevier’s Pathway Studio (version 11.0//Elsevier), all relationships between the differentially expressed proteins among all conditions were depicted based on the Ariadne ResNet (22) For proteins identified in the shotgun analysis, the Subnetwork Enrichment Analysis (SNEA) algorithm was used to detect the statistically significant altered biological pathways in which the identified proteins are involved. This algorithm uses Fisher’s statistical test to detect any non-random associations between two categorical variables organized by a specific relationship. Also, this algorithm starts by creating a central “seed” from all the relevant identities in the database and builds connections with associated entities based on their relationship with the seed. SNEA compares the sub-network distribution to the background distribution using one-sided Mann-Whitney U- Test and calculates a p-value; thus, representing a statistical significance between different distributions. In all analyses that we performed, the GenBank ID was used to form experimental groups based on the different conditions present for analysis. The pathway networks were reconstructed based on biological processes and molecular functions for every single protein, along with its associated targets.

### Mutation Identification

Protein identification was also performed using the mutation-specific database.32 XMan v2 database contains 2 539 031 mutated peptide sequences from 17 599 Homo sapiens proteins (2 377 103 are missense and 161 928 are nonsense mutations). The interrogation was performed with Proteome Discoverer 2.3 software and Sequest HT package, using an iterative method. The precursor mass tolerance was set to 15 ppm and the fragment mass tolerance was set to 0.02 Da. For high confidence results, the false discovery rate (FDR) values were specified to 1%. A filter with a minimum Xcorr of 2 was applied. The generated result file was filtered using a Python script to remove unmutated peptides. All mutations were then manually checked based on MSMS spectra profile. The structure of mutated proteins was constructed with PremPS (https://lilab.jysw.suda.edu.cn/research/PremPS/) (23).

### Druggable Genome Database and Clinical Trials

To identify drug candidates targeting the surfaceome proteins specific to GBM, the relationship between protein-coding genes and drugs was analyzed from different sources including Drug Central (https://drugcentral.org), DrugBank (https://go.drugbank.com/), ApexBio (https://www.apexbt.com/), DGIdb (https://www.dgidb.org/), FDA Approved Drugs (https://www.accessdata.fda.gov/scripts/cder/daf/), ClinicalTrials.gov, and/or PharmGKB (https://www.pharmgkb.org/).

To determine whether drug candidates were investigated in therapeutic trials for GBM patients, ClinicalTrials.gov web-based resource was used (https://clinicaltrials.gov/ct2/home). This resource provides information on publicly and privately supported clinical studies on a wide range of diseases and conditions and is maintained by the National Library of Medicine at the National Institutes of Health (US). Information on ClinicalTrials.gov is provided and updated by the sponsor or principal investigator of the clinical study. Among the clinical trials conducted in patients, children, and adults, with glioblastoma, the search was conducted among the trials corresponding to interventional studies with a status recruiting, not yet recruiting, active, not recruiting, enrolling by invitation, or completed, which represented a list of 1750 clinical trials.

### Patient Samples and Consent

Patients with newly diagnosed glioblastoma were prospectively enrolled between September 2014 and November 2018 at Lille University Hospital, France. Patients were adult, had no medical history of other cancers or previous cancer treatment, no known genetic disease potentially leading to cancer and no neurodegenerative disease. Tumors samples were processed within 2 hours after sample extraction in the surgery room to limit the risk of degradation of proteins. Characteristic of the cohort has been published in (24) (**Supplementary Table 1**). Approval was obtained from the research ethics committee (ID-RCB 2014-A00185-42) before initiation of the study. The study adhered to the principles of the Declaration of Helsinki and the Guidelines for Good Clinical Practice and is registered at NCT02473484. Informed consent was obtained from patients.

## Results

**Figure 1:**
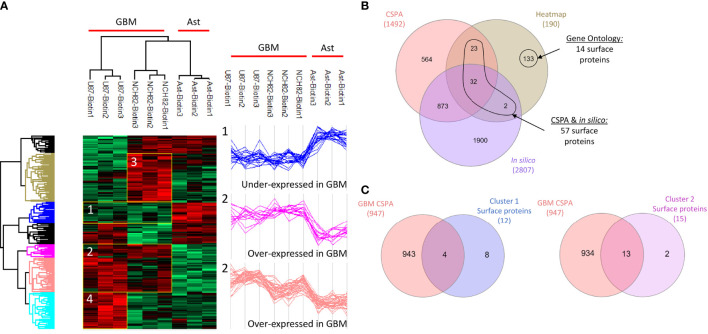
Healthy astrocyte cell line, U87 and NCH82 GBM cell lines were biotinylated and lysed. Biotinylated surface proteins were purified on streptavidin beads, digested, and analyzed with LC-MS/MS. MaxQuant and Perseus software were used for the statistical analysis. **(A)** A heatmap was generated to show proteins with expression significantly different between cell lines. Two clusters are highlighted (1 and 2). **(B)** Venn diagram was performed between the 190 proteins of the heatmap and the surface proteins from CSPA and in silico CSPA databases to identify 57 surface proteins. Analysis of Gene Ontology of the different proteins allowed the discovery of 14 additional surface proteins. **(C)** Proteins from cluster 1 and cluster 2 were compared to surface proteins described for different GBM cell lines in CSPA (GBM CSPA).

Malignant cells adopt a complete proteomic makeover, especially on their surface. Surfaceome study allows emphasizing the changes in the interactions between cells and with their environment. Here, we compared the surfaceome of two different human glioblastoma cell lines, U87 and NCH82, with a human astrocyte cell line. Cell-surface proteins were labelled and captured with membrane-impermeable EZ-Link Sulfo-NHS-SS-Biotin from intact cells. Shotgun proteomic analysis of these three cell lines yielded 2,920 protein identifications across all the samples (**Supplementary Data 1**). We applied a filter to retain only the proteins found in at least two of the three replicates. Then, the variations in protein expression were analyzed between the three cell lines. As a criterion of significance, we applied an ANOVA test with a significance threshold of p < 0.05. A heatmap was created from which 190 proteins showed a significant difference in LFQ expression between astrocytes, NCH82, and U87 cell lines (**Figure 1A**, **Supplementary Data 2**, **3**). Surface proteins were then identified by cross-checking the total list of heatmap proteins with the lists of surface proteins provided by the cell surface protein atlas (CSPA) (13) and the list of predicted surfaceome proteins (14). Gene Ontology enrichment allowed the identification of some additional surface proteins. We found 57 surface proteins thanks to CSPA and *in silico* databases and 14 additional proteins being described as membrane proteins for a total of 71 surface proteins with differential expression between the three cell lines (**Figure 1B**). Four clusters were highlighted, cluster 1 regroups under-expressed surface proteins in the two GBM cell lines and cluster 2 overexpressed proteins (**Figure 1A**). The cluster 3 represents NCH82 over-expressed proteins and the U87 over-expressed proteins are in the cluster 4 (**Figure 1A**).

### Over-Expressed and Exclusive Surface Proteins in Immortalized Astrocyte Cell Line

**Figure 2:**
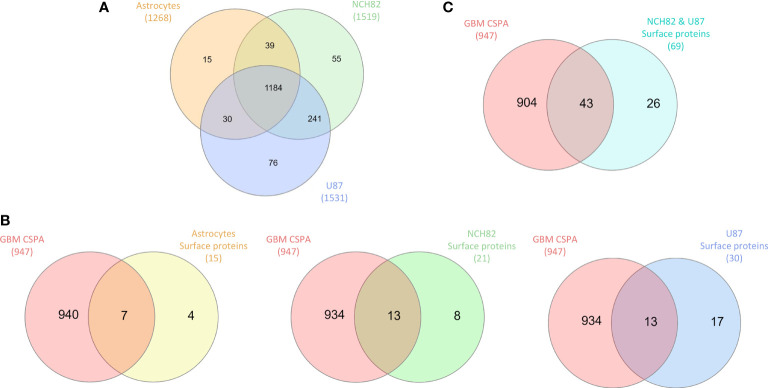
**(A)** Proteins identified in all cell lines are compared in Venn diagram to highlight exclusive proteins to each cell line and common proteins to GBM cell lines. **(B)** Exclusive surface proteins from each cell line were compared to surface proteins from GBM cell lines in CSPA (CSPA GBM). **(C)** Common proteins to NCH82 and U87 GBM cell lines were compared to surface proteins from GBM cell lines in CSPA (CSPA GBM).

We were first interested in the proteins under-expressed or unexpressed in GBM cells compared to astrocytes. On the heatmap (**Figure 1A**), cluster 1 includes 21 proteins that are under-expressed in NCH82 and U87 compared to the astrocyte cell line (**Supplementary Data 2**). Among these proteins, 12 are known to be expressed at the plasma membrane. We compared these proteins to the surface proteins described in GBM cell line and primary culture in CSPA (GBM CSPA) database. Four proteins were found in GBM CSPA databases and 8 additional proteins were found in astrocyte cell line (**Figure 1C**, **Supplementary Data 2**). Among these proteins, we found Cell adhesion molecule 3 (*cadm3*) involved in intercellular adhesion and Fibrillin-2 (*fbn2*), an extracellular matrix component. Stomatin (*stom*) which regulates ion transport was also found in this cluster of under-expressed surface proteins. We then examined the proteins exclusive to each cell line and among the 15 proteins only expressed by astrocytes cell line, 11 were membrane-bound and so, unexpressed in GBM cell lines (**Figure 2A**). 7 of these 11 surface proteins are described in GBM CSPA databases (**Supplementary Data 4**, **Figure 2B**). In addition to these proteins, we also found 4 more cell surface proteins not described in the lists above (**Supplementary Data 4**). Among these proteins, we found the desmosomal protein plakoglobin (*jup*) which plays a central role in the structure and function of submembranous plaques (25). We have also identified NHE-RF1 (*slc9a3r1*), known to be localized to the plasma membrane in normal astrocytes and showing a cytoplasmic shift within GBM tumor cells (26).

### Over-Expressed and Exclusive Surface Proteins in GBM Cell Lines

Overexpressed and exclusive proteins to GBM cell lines were abundant in our data. Indeed, cluster 2 of the heatmap includes 47 proteins overexpressed in GBM cell lines compared to astrocytes cell line. Among these proteins, we counted 15 proteins described to localize at the plasma membrane including 13 proteins already described in GBM CSPA and 2 additional surface proteins (**Figure 1C**, **Supplementary Data 2**). Some of these membrane proteins are involved in tumor progression such as Neurogenic locus notch homolog protein 2 (*notch2*) (27), Aspartyl/asparaginyl beta-hydroxylase (*asph*) (28) and Procathepsin L (*ctsl*) for which high expression is unfavorable in glioma (29). We also found the proteins Prostaglandin F2 receptor negative regulator (*ptgfrn*) and Collagen alpha-1(VI) chain (*col6a1*), which correlate with a poor prognosis in GBM (30, 31). Two more clusters of over-expressed proteins in each GBM cell line are highlighted. In the cluster 3, we found 15 surface proteins over-expressed in NCH82 cell line (**Figure 1A**) in which 5 are not described in GBM CSPA (**Supplementary Data 2**). Finally, cluster 4 contains 10 surface proteins over-expressed in U87 cell line among which one protein is not found in GBM CSPA (**Figure 1A**, **Supplementary Data 2**). We have also identified 21 surface proteins only expressed by NCH82 among the 55 proteins identified (**Supplementary Data 4**, **Figure 2B**). Among these cell surface proteins, 13 were already described in GBM CSPA (**Supplementary Data 4**).

We identified 8 additional surface proteins in NCH82 cell line. Among these proteins we found, Guanylate-binding protein 1 (*gbp1*) and Pro-neuregulin-1 (*nrg1*) which are key players in glioblastoma progression (32, 33). We have also identified Raftlin (*rftn1*) which is involved in the cell entry of poly(I:C), ligand of TLR3 (34). It could therefore be a target in therapies involving TLRs (35).

Regarding the 76 proteins only expressed by U87 cell line, 30 surface proteins have been identified (**Supplementary Data 4**, **Figure 2B**). 13 of these proteins are found in GBM CSPA. We have also identified 17 other proteins that are described as membrane-bound in the literature (**Supplementary Data 4**). Among these proteins, we found Calpain-5 (*capn5*) which is already described in surgical biopsies of glioblastoma (36). We also found Paxillin (*pxn*) which is associated with a poor prognosis of glioblastoma (37). Sodium-dependent phosphate transporter 1 (*slc20a1*) is described to be over-expressed in high grade gliomas (38) and Integrin-linked protein kinase (*ilk*) to promote glioblastoma invasion (39). Finally, Caveolin-1 (*cav1*) could serve as a biomarker to predict response to chemotherapy. Indeed, its over-expression seems to confer sensitivity to the most commonly used chemotherapy for glioblastoma, temozolomide (40). Moreover, 69 proteins described as membrane proteins were found among the 241 proteins common to both NCH82 and U87 glioblastoma cell lines and not expressed by astrocyte cell line (**Supplementary Data 4**, **Figure 2C**). Among these proteins, 43 are found in the GBM CSPA database. We found 26 additional surface proteins that are not included in the lists of surface proteins previously mentioned (**Supplementary Data 4**). Among them, we found Glioma pathogenesis-related protein 1 (*glipr1*), Receptor of activated protein C kinase 1 (*rack1* or *gnb2l1*), Protein disulfide-isomerase (*p4hb*), and Carbonic anhydrase 9 (*ca9*) known to be over-expressed in glioblastoma and to promote tumor growth (41–44). We also find Microtubule-actin cross-linking factor 1 (*macf1*) recently described as a novel radiosensitization target in glioblastomas (45) and Protein NDRG1 (*ndrg1*) which is over-expressed in glioma resistant to antiangiogenic therapy (46).

### Most of GBM cell Surface Proteins Are Linked to Tumor Growth and Immune Regulation

**Figure 3:**
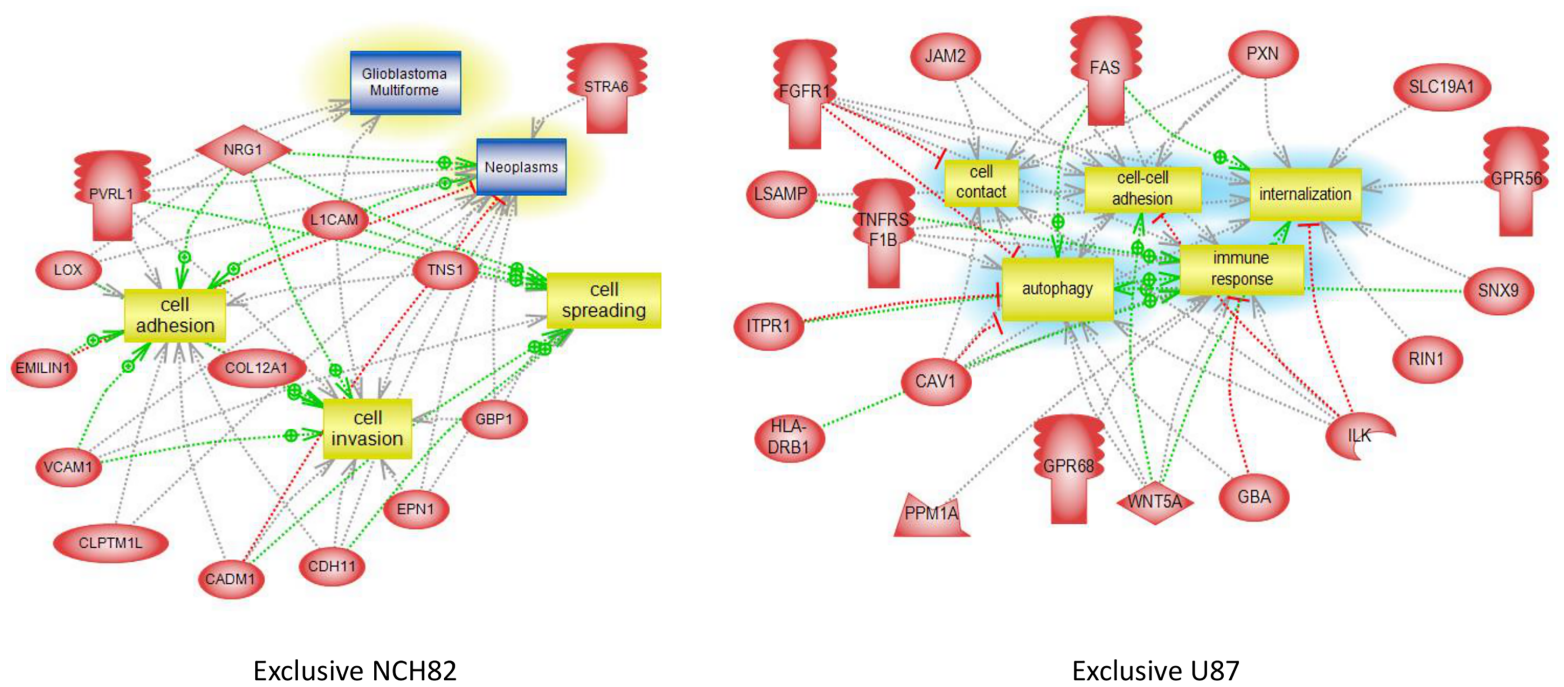
Global pathway analyses of proteins only expressed by NCH82 and U87 GBM cell lines.

As described before, several surface proteins identified in GBM cell lines are involved in tumor growth. Biological pathways analyses highlighted several proteins only expressed by NCH82 or U87 cell line linked to cell invasion or cell spreading, two key processes for GBM development and drug resistance (**Figure 3**, **Supplementary Figure 1**). Moreover, most of the cell surface proteins are related to cell contact and cell adhesion pathway, another main process for tumor growth (**Figure 3**, **Supplementary Figure 1**). The two GBM cell lines also over-expressed surface proteins linked to the vascularization and proliferation pathways (**Supplementary Figure 1**). Biological pathways analyses also established that several common proteins for GBM cell line and proteins only expressed by U87 (**Figure 3**) are involved in immune regulation processes such as negative regulation of CD8-positive, alpha-beta T cell activation (*hfe*), negative regulation of activated T cell proliferation (*pdcd11g2, cd274*) or negative regulation of interferon-gamma production (*c1qbp*).

### Expression of Mutated Surface Proteins in the Glioblastoma Cell Lines

**Figure 4:**
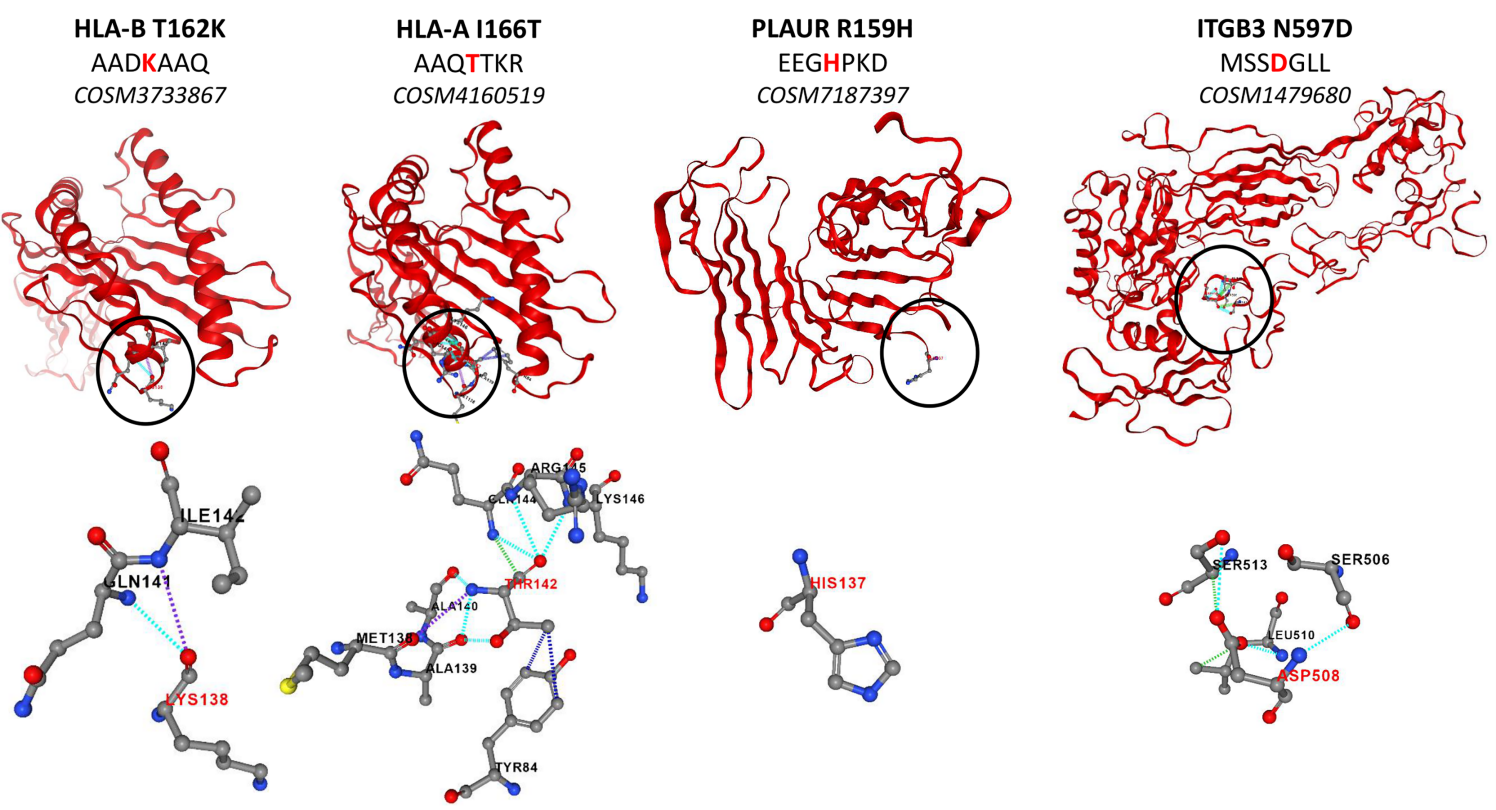
Structure of mutated proteins according to PremPS.

Several studies have reported the high number of protein mutations related to tumor progression in GBM (47–49). In this context, we investigated the mutations of surface proteins in GBM cell lines. For that purpose, we used a human database combined with the XMan v.2 database (15). This database contains information about mutated peptides that can be found in some cancers, extracted from the COSMIC database. After applying statistic filters, 114 mutations were identified. The MSMS spectra of these 114 mutations were manually checked for specific fragmentation of the mutated amino acid. Thus, thirty-three mutated peptides were identified among which 9 are only detected in both NCH82 and U87 GBM cell lines compared to astrocyte cell line. Among the proteins from which mutated peptides were identified, we retrieved RELT-like protein 1 (*rell1*), Cytochrome b-245 light chain (*cyba*), Epidermal growth factor receptor (*egfr*) and Cytochrome b reductase 1 (*cybrd1*) for which missense mutations are found in their intracellular domains. Other proteins mutated this time in their extracellular domain have been identified such as Urokinase plasminogen activator surface receptor (*plaur*), Integrin beta-3 (*itgb3*), and different subunit of HLA class I and II histocompatibility antigen such as B-41 alpha chain (*hla-b*), A-24 alpha chain (*hla-a*) and DP beta 1 chain (*hla-dpb1*) (**Figure 4**).

### Tracking Identified Surface Proteins in Patients

In this study, proteomic analyses were performed on three different replicates of cell samples. Some variation in the protein expression can be observed between replicates of a same cell sample (**Figure 1A**). Since the number of replicates was limited, we wanted to validate the expression in GBM patients of all the surface proteins identified in this study. To do so, we compared the proteins of each heatmap clusters (**Figure 1A**, **Supplementary Data 2**) as well as the proteins exclusive to each cell line and the proteins common to both NCH82 and U87 cell line (**Figure 2A**, **Supplementary Data 4**) to the ones identified in patient’s glioblastoma using spatial proteomic guided by MALDI-mass spectrometry (24). Characteristics of the GBM cohort are presented in (**Supplementary Table 1**). It has allowed to identify 66 of our surface proteins expressed in at least 70% patient’s GBM from this cohort.

Of all the surface proteins we identified in this study (**Supplementary Data 2** and **4**), 67 are not described in the GBM cell lines in the CSPA database. It was therefore necessary to confirm the expression of these additional surface proteins in patient’s GBMs. Thus, we can confirm the expression of 32 surface proteins in 70% of the GBM patients cohort (24) among the 67 additional proteins not founded in the GBM CSPA data (**Supplementary Data 2** and **4**). Finally, among the surface proteins identified in the GBM cell lines and in the patient’s GBM we found 5 proteins not described in healthy brain tissues according to human protein atlas (**Table 1**, **Supplementary Table 3**). In which we have HSPD1 already used in clinical trials as described before and LGALS3BP which have no known drug interaction yet. The two proteins are associated with a bad overall survival (**Supplementary Figure 2**).

**Table 1:** Expression of surface proteins in GBM patients.

Protein Ids	Gene names	Protein names	Astrocytes	NCH82	U87	Glioma patients
P12109	**COL6A1**	Collagen alpha-1(VI) chain	0	X	X	49/50 (24)
P12110	**COL6A2**	Collagen alpha-2(VI) chain	0	X	X	39/50 (24)
P12111	**COL6A3**	Collagen alpha-3(VI) chain	0	X	X	50/50 (24)
Q08380	**LGALS3BP**	Galectin-3-binding protein	0	X	X	45/50 (24)
P10809	**HSPD1**	60 kDa heat shock protein, mitochondrial	0	X	X	50/50 (24)
P30479	**HLA-B**	HLA class I histocompatibility antigen, B-41 alpha chain *Mutated for T162K*	0	0	X	7/17 (50)
P05534	**HLA-A**	HLA class I histocompatibility antigen, A-24 alpha chain *Mutated for I166T*	0	0	X	4/17 (50)

Moreover, the mutation of HLA class I histocompatibility antigen A and B are also found in several patient’s GBM (50) (**Table 1**).

### Clinical Investigation for the Selected Specific Glioblastoma Targets

We selected 78 surface proteins only found in each GBM cell lines (**Figure 2C**) or overexpressed (**Cluster 2,** **Figure 1A**) in both NCH82 and U87 GBM cell lines (**Supplementary Table 2**) and the 9 mutated surface proteins only expressed by NCH82 or U87. We analyzed the expression of these proteins inside healthy tissues and cancers thanks to Human Protein Atlas and Catalogue Of Somatic Mutation In Cancer (COSMIC) databases. Thus we highlighted 25 proteins not expressed in healthy brain tissues (**Supplementary Table 3**). The druggability of the selected surface proteins specific to GBM was assessed using drug and compound databases as described in methods. Our approach identified among targetable proteins CPM, CYBA, EGFR, HLA-A, HLA-B, HSPD-1, P4HA2 which were druggable with either chemotherapy, tyrosine kinase inhibitors, vaccines, anti-CTLA4 or vitamins. A non-exhaustive list of clinical studies detailing the trials investigating the antitumor efficacy of these strategies in GBM patients is in **Supplementary Table 3**. These proteins also had interactions with other drugs or compounds not yet under clinical investigation such as antiepileptics to target CPM or HLA, lipid-lowering drugs to target CYBA, or antibiotics to target HSPD1, thus showing the potential for drug repurposing to treat GBM. Moreover, our approach identified C1orf159, CD151, and LGALS3BP which have no known drug interaction yet.

## Discussion

In this work, we compared the surfaceome of glioblastoma cell lines to an immortalized astrocyte cell line. We were able to highlight the different changes in the surface proteins expressed in cancer cells. Among these changes, we found an under-expression of proteins involved in cell adhesion in glioblastoma cell lines compared to immortalized astrocytes. Indeed, NCH82 and U87 underexpress proteins such as CADM3, CADM4, or NRCAM involved in intercellular adhesion. Impairment of cell-cell adhesion is one of the processes allowing tumor escape and metastasis. Alteration of adhesion protein expression is a common event in many types of cancer. Many of these proteins are being studied as potential biomarkers or therapeutic targets (51). The extracellular matrix organization and integrity is another crucial aspect of the metastasis process. Several proteins related to the extracellular matrix organization are overexpressed in GBM cell lines compared to astrocytes cell line. These results suggest a complete extracellular matrix reorganization by cancer cells. On the other hand, some surface proteins overexpressed in GBM cells compared to immortalized astrocytes are involved in the generation of metabolic energy. The immune system regulation by cancer cells can also be investigated by surfaceome study. Indeed, some proteins expressed only in GBM cells are involved in the negative regulation of the immune response. Moreover, we highlight changes in protein localization within cancer cells. NHE-RF1 previously described as showing a cytoplasmic shift within GBM tumor cells compared to healthy cells (26), is only found in the membrane of immortalized astrocytes. Thus, this protein known to be membrane-bound shows intracellular localization in glioblastoma cells. This suggests that commonly intracellular proteins may be expressed at the cell surface within cancer cells. In fact, protein mislocalization is known to be a less emphasized mechanism in cancer (52). We also identified several mutations in surface proteins only expressed by GBM cell lines. Among these mutated proteins we find RELT-like protein 1 (*rell1*), Cytochrome b-245 light chain (*cyba*), Epidermal growth factor receptor (*egfr*), and Cytochrome b reductase 1 (*cybrd1*). These mutations are found in the cytoplasmic domain and could alter the signaling pathways in which these proteins are involved. Thus, we have demonstrated that the study of surface proteins allows us to highlight different processes used by cancer cells to modify their environment and metabolism to increase tumor growth. It is a new tool to explore specific tumor development mechanisms through a non-traditional approach.

The surfaceome study is a useful tool for fundamental research to understand the mechanisms of tumor growth. It also enables the discovery of new biomarkers for clinical purposes. Indeed, in this data, we found 78 surface proteins over-expressed or exclusive to GBM cell lines compared to astrocyte cell lines (**Cluster 2,** **Figures 1A**, **2C**, and **Figure 2C**). Among these proteins, 28 are not described in GBM CSPA database. It may be an opportunity to complete databases after localization validation of these proteins with the literature. On the other hand, we screened the human protein atlas for these 78 surface proteins to highlight potential therapeutic targets for immunotherapy. Thus, 25 surface proteins showed low expression on brain-healthy tissues (**Table S1**, proteins highlighted in blue), and 6 of these proteins are described in GBM tissues (**Table S1**, proteins highlighted in purple) according to the human protein atlas. Moreover, among mutated surface proteins identified, some are mutated in their extracellular domain (**Table S1**, proteins highlighted in red). Among these proteins we found Urokinase plasminogen activator surface receptor (*plaur*), Integrin beta-3 (*itgb3*) and the different subunits of HLA class I and II histocompatibility antigen such as B-41 alpha chain (*hla-b*), A-24 alpha chain (*hla-a*) and DP beta 1 chain (*hla-dpb1*). These 5 proteins present extracellular mutations unique to GBM cell lines and described in the literature in very few cancers. As it is the case for mutated HLA-B and HLA-A previously described within GBM tissues (50). Thus, we identified 11 surface proteins which could be used as a therapeutic target in immunotherapy against GBM. Among these 11 targets, 7 are already used in clinical trials (**Table S2**), for example, HSPD1 is also described in GBM tissues (24). Moreover, C1orf159, CD151, LGALS3BP, and mutated proteins described above are not yet used in clinical trials but worth interest, notably LGALS3BP also found in GBM tissue (24) like mutated HLA-A and HLA-B (50). We also identified from spatial proteomic studies of 50 GBM patients, twelve proteins in common with our data including LGALS3BP and HLA-A2. Nine of the twelve have been associated with worse overall survival (50). LGALS3BP is included in the nine proteins which is in line with other data observed in breast cancer. LGALS3BP inhibits the differentiation of monocyte-derived fibrocytes through CD209/SIGN-R1 in mouse spleen and is secreted from breast cancer for metastasis (53).

Taken together, our data suggest that these surface proteins could be candidate targets for alternative therapeutic strategies such as new CARs against mutated GBM-specific proteins, which would reduce the immune response and off-target effect. This work showed that exploring the surfaceome of GBM cells has the potential to identify new therapeutic targets including mutated surface proteins exclusive to GBM. However, it is important to be aware that these results are derived from surfaceome analyses of immortalized cell lines in a limited range of replicates. The cross-validation of our results with GBM patients proteomics data and with the Human Protein Atlas data helped us to select the more specific potential targets which still need to be validated on a more relevant study model such as patients’-derived organoids and an increased number of replicates. Using patients’ derived tumoroids in order to select the right antigens expressed by the tumor cells could help to translate this kind of approach into the clinic in order to perform personalized therapies.

## Data Availability Statement

Proteomics data including MaxQuant files and annotated MS/MS have been deposited to the ProteomeXchange Consortium *via* the PRIDE partner repository with the dataset identifier PXD027110.

## Ethics Statement

The studies involving human participants were reviewed and approved by ID-RCB 2014-A00185-42. The patients/participants provided their written informed consent to participate in this study.

## Author Contributions

Conceptualization, IF and MS. Methodology, MR. Software, FK, NH, MR, TC, and SA. Validation, MR, TC, and SA. Formal analysis, MR and MS. Investigation, IF, NH, MD, MR, and MS. Resources, IF and MS. Data curation, MR. Writing—original draft preparation, MR and MS. Writing—review and editing, IF, FK, MD, MR, and MS. Visualization, MR. Supervision, IF and MS. Project administration, MD and MS. Funding acquisition, IF, NH, MS, and TC. All authors contributed to the article and approved the submitted version.

## Funding

This research was funded by Institut national de la Santé et de la recherche Médicale (Inserm), Canceropole Nord.

## Conflict of Interest

The authors declare that the research was conducted in the absence of any commercial or financial relationships that could be construed as a potential conflict of interest.

## Publisher’s Note

All claims expressed in this article are solely those of the authors and do not necessarily represent those of their affiliated organizations, or those of the publisher, the editors and the reviewers. Any product that may be evaluated in this article, or claim that may be made by its manufacturer, is not guaranteed or endorsed by the publisher.

## Supplementary Material

Supplementary Table 1 Expression in healthy and cancers tissues of surface proteins over-expressed or exclusive to GBM cell lines according to Human Protein Atlas.

Supplementary Table 2 Drug candidates targeting genes specific to glioma and existing therapeutic clinical trials in glioma patients.

Supplementary Data Sheet 1 Raw data before the application of any filters.

Supplementary Data Sheet 2 Proteins list for the clusters of the heatmap **Figure 1**.

Supplementary Data Sheet 3 p-value of proteins presented on the heatmap.

Supplementary Data Sheet 4 Exclusive proteins list for each cell line.

Supplementary Figure 1 Global pathways analyses for clusters 1, 2, 3 & 4.

Supplementary Figure 2 Overall survival studies and HIS results for LGALS3BP and HSPD1.
