# Supplementary figures and images for: Surfaceome Proteomic of Glioblastoma Revealed Potential Targets for Immunotherapy
Source: Front Immunol. 2021 Sep 27;12:746168. doi: 10.3389/fimmu.2021.746168 (PMC8503648; doi:10.3389/fimmu.2021.746168)

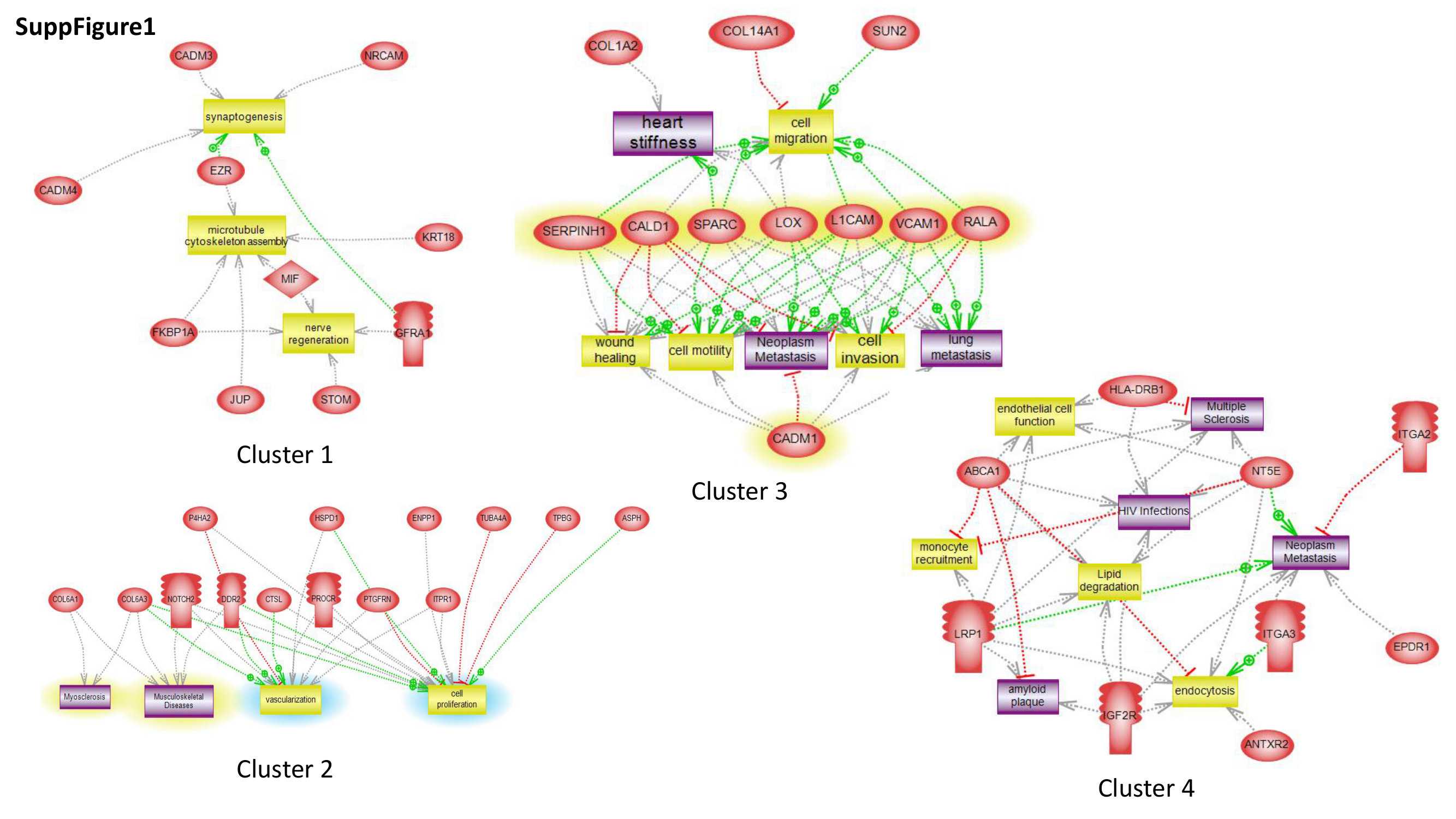

Supplement: Supplementary Figure 1 — Global pathways analyses for clusters 1, 2, 3 & 4. [file Image_1.tiff]

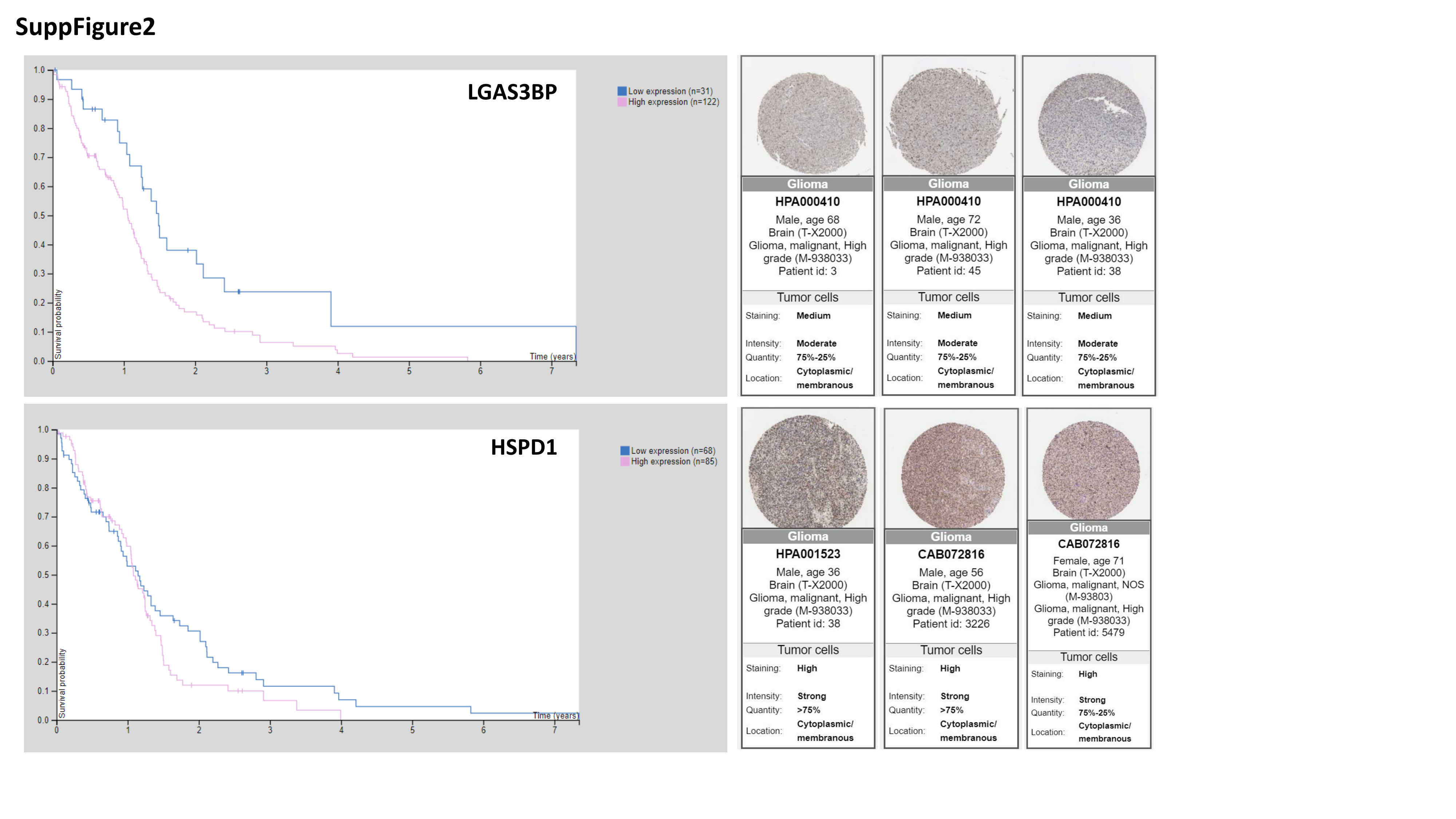

Supplement: Supplementary Figure 2 — Overall survival studies and HIS results for LGALS3BP and HSPD1. [file Image_2.tif]
